# To include or not include: renal dialysis policy in the era of universal health coverage

**DOI:** 10.1136/bmj.m82

**Published:** 2020-01-28

**Authors:** Yot Teerawattananon, Saudamini Vishwanath Dabak, Levina Chandra Khoe, Diana Beatriz S Bayani, Wanrudee Isaranuwatchai

**Affiliations:** 1 Health Intervention and Technology Assessment Program (HITAP), Ministry of Public Health, Nonthaburi, Thailand; 2 Saw Swee Hock School of Public Health, National University of Singapore, Singapore; 3 Department of Community Medicine, Faculty of Medicine, Universitas Indonesia, Jakarta, Indonesia; 4 Health Technology Assessment Unit, Department of Health, Manila, Republic of the Philippines; 5 St Michael’s Hospital, Li Ka Shing Knowledge Institute, Centre for Excellence in Economic Analysis Research (CLEAR), Toronto, Ontario, Canada

## Abstract

Expensive treatments such as renal dialysis are a challenge for countries aiming for universal coverage. **Yot Teerawattananon and colleagues** set out a systematic approach to ensuring it is affordable

End stage renal disease (ESRD) is a fatal condition that affects 10 million people annually. Although effective treatment exists, coverage remains low. In 2010, only 27% of those in need of renal replacement therapy globally actually received it, with the proportion being between 2% and 5% in low and lower middle income countries.^1^ The rise in the ageing population coupled with increasing prevalence of diabetes and hypertension and other non-communicable diseases in low and middle income countries is expected to exacerbate the situation. In addition to investing in preventive interventions, countries will need to determine how best to provide expensive treatment. This is particularly challenging within the context of implementing universal health coverage (UHC).^2^ Policy makers need to ensure that their UHC programmes are ethical and make best use of resources. 

 We examine the challenges of implementing universal access to renal replacement therapy and offer a guide to help policy makers navigate the decision making process. 

## The problem: limited access to expensive care 

Dialysis costs are unaffordable for most people in low and middle income countries. A systematic review suggests that the annual direct cost of dialysis for one patient equals the average income of three people in an upper middle income country.^3^ The situation is worse in lower middle income countries, where six people’s income would be needed to cover the cost of a year’s dialysis for one patient; in a least developed country, the cost is equivalent to the average income of 12 people.^3^ These cost estimates explain why ESRD is a leading cause of catastrophic health expenditure and impoverishment worldwide.

ERSD can be treated with kidney transplantation or renal dialysis.^4^ Renal transplantation is the only definitive treatment, but access is limited because of low levels of organ donation or lack of infrastructure to support transplantation. The high upfront cost associated with renal transplantation can be a barrier for extending coverage to all patients, particularly in low and middle income countriess.^5^ Renal dialysis is therefore the most viable option for patients around the world. There are two options available for renal dialysis, haemodialysis and peritoneal dialysis, and health outcomes are similar for both.^6^ Haemodialysis is used most often but incurs high costs and is largely provided in centres in large cities; peritoneal dialysis is generally more cost effective and scalable as it is mainly carried out at home and has lower infrastructural requirements.^7^

## Potential solutions

Economic growth in South East Asia has resulted in improved living conditions and longer life expectancy for its population.^8^ ^9^ Indonesia, the Philippines, and Thailand have all included renal dialysis in their UHC programmes but implemented it in different ways. The challenge of providing universal renal dialysis is particularly acute given resource constraints in the three settings, as income per capita ranges from $2900 (£2200; €2600) to $6600.^9^

Thailand did not include renal dialysis in its benefits package when the UHC programme was initiated in 2002 as it would have bankrupted the programme before it had even begun. Under pressure from civil society and professional groups, the Thai government commissioned studies to understand the situation for ESRD in the country and explored possible solutions to support households incurring debilitating costs to treat this disease. Researchers from the Thai Ministry of Public Health conducted a study that informed implementation of a cost effective policy for universal coverage. The policy dictated starting with peritoneal dialysis as opposed to free choice or haemodialysis first.^4^ This intervention was supplemented with other policies to incentivise healthcare providers to offer peritoneal dialysis, such as training nurses to administer peritoneal dialysis to patients, developing an extensive delivery system to provide the dialysis solution directly to patients’ homes, and charges to discourage patients with ESRD who are medically fit for peritoneal dialysis from opting for haemodialysis.^10^ Even though Thailand had mainly used haemodialysis, it has been able to move to a system dominated by peritoneal dialysis in the 10 years since renal dialysis was added to the UHC benefits package.^11^

Indonesia and the Philippines both included ESRD in their benefits packages at the start of their UHC programmes, although without any policy assessment of the financial implications. Patients in these two countries can choose any dialysis option free of charge, but doctors commonly advise them to start with in-centre haemodialysis. In Indonesia, renal dialysis is fully covered by the Jaminan Kesehatan Nasional (JKN), which is one of the largest single payer health insurance schemes, covering 203 million people in 2018.^12^ The Philippines’ scheme provides only partial coverage of haemodialysis, covering 90 of the 156 sessions normally required each year with the expectation that the remaining sessions would be paid for by patients. A recent study found that most patients have not been able to bear the cost of the remaining sessions. Consequently, patients have been forced to take a suboptimal number of haemodialysis sessions, lowering their quality of life and shortening their life expectancy by around eight years compared with dialysis patients in Indonesia and Thailand.^13^

Although dialysis was provided under Indonesia’s UHC scheme, treatment remains elusive for a large proportion of the population. A study estimated that only 13% of ESRD patients had access to renal dialysis services in Indonesia because only 10 of the 6000 inhabited islands in Indonesia have a haemodialysis centre.^14^ There are even fewer peritoneal dialysis centres on these islands. The coverage of renal dialysis is also low in the Philippines (22%),^15^ whereas Thailand manages full coverage.^16^

In all three countries, renal dialysis was among the highest claim items in their national UHC programmes. The amount spent on renal replacement therapy, as a percentage of the total UHC budget, was 4.16% of $1.3bn in Indonesia, 7.7% of $1.9bn in the Philippines, and 5.1% of $5.2bn in Thailand. This is a high expenditure on one disease since dialysis patients constitute only a small proportion of the UHC beneficiaries (0.03% (63 818 patients) in Indonesia, 0.06% (61 763) in the Philippines, and 0.11% (53 234) in Thailand).^16^ ^17^ ^18^

## Policy choices for low and middle income countries

Inclusion of renal dialysis in UHC benefits packages poses a challenge to many countries and requires both political and financial commitment. Many countries are seeking to implement universal dialysis, including Pakistan, Nigeria, Samoa, and India, which has announced it is setting up dialysis centres in around 700 districts and offering free haemodialysis to people below the poverty line.^19^ ^20^ ^21^

Based on our experience supporting the government implementation of renal dialysis policies in Indonesia, the Philippines and Thailand, we set out the policy choices for countries, particularly low and middle income, that are trying to provide universal renal replacement therapy to their citizens. Policy makers can use the questions below to drive the discourse on renal replacement therapy under UHC and to weigh the advantages and limitations of each policy option and build consensus among stakeholders.

The first question is whether renal replacement therapy should be included in the UHC benefits package. If the response to that question is “no” or “not for now,” the country must come to terms with the persistence of catastrophic health expenditure in the population resulting from ESRD.

Should the country decide to include renal replacement therapy the next decision is which types of treatment should be covered. Pre-emptive kidney transplantation has been shown to provide the best value for investment in many settings.^2^ However, universal access requires availability of sufficient kidney donors in the country and the capacity for providing transplantation and subsequent care. If these two conditions are not met, as was the case in Indonesia, the Philippines, and Thailand, the country has to decide whether to adopt peritoneal dialysis or haemodialysis as the first line of treatment and how this will be implemented.

If the country decides to opt for peritoneal dialysis, it will have to consider patient and provider acceptability as well as the need for a support system in the community since the treatment is largely carried out by patients at home. 

If the country opts for haemodialysis first or through a free choice policy, then it has to decide between centre or home based treatment. For in-centre haemodialysis, the country needs to ensure there is sufficient infrastructure available, including haemodialysis machines and accessibility to haemodialysis centres for patients, and a mechanism to ensure adequate treatment is affordable to avoid suboptimal dialysis. The most advanced option is home based treatment, but this requires investment in haemodialysis machines for each patient as well as training of patients and care givers.

None of these options are cheap. Kidney transplantation requires large, upfront investment, even though it is the cheapest and most definitive treatment option in the long term. Peritoneal dialysis is the second cheapest and relatively more cost effective option, with direct medical costs being about 70-80% of those for in-centre haemodialysis.^3^ Home based haemodialysis is the most expensive treatment option. When a pre-emptive kidney transplantation policy is not viable, offering peritoneal dialysis as the first treatment is the most feasible and scalable option, particularly in low and middle income countries, because it has lower infrastructural requirements. The benefits package should also include effective preventive measures, such as controlling diabetes and hypertension, and workforce training to ensure sustainable management of these high cost treatments.^2^ ^5^

The experiences of Indonesia, the Philippines, and Thailand suggest that universal renal replacement therapy is a hard but necessary choice for countries to make on their journey towards UHC. Indeed, UHC policies in countries will be judged by how they respond to the needs of patients with ESRD, using it as a barometer for a society’s willingness to pay for its healthcare.^5^ A systematic approach to decision making on treating ERSD and other expensive conditions will help ensure that countries make best use of their resources.

**Summary points**

- End stage renal disease (ESRD) is a life threatening condition that results in most patients requiring a lifetime of high cost renal dialysis
- Governments have grappled with the political, economic, and ethical challenges of providing treatment with their available budgets
- This tension is more prominent in resource limited countries that have committed to universal health coverage
- A systematic approach to inform universal renal dialysis policy can provide feasible, affordable, and scalable care
- Providing peritoneal dialysis as first line of treatment is the most feasible option for low and middle income countries

## Notes

Contributors and sources: All authors work on health technology assessment (HTA) and health systems and policy research. YT is the founder of and senior researcher at the Health Intervention and Technology Assessment Program (HITAP) in the Ministry of Public Health, Thailand, and is a globally recognised leader in the field of HTA; he has led HITAP in supporting the development of HTA in the South-east Asia Region as part of the International Decision Support Initiative (iDSI). SD is manager of HITAP’s International Unit and has conducted health systems research in the South-east Asia region. LCK has worked on studies conducted by the HTA Committee in Indonesia. DB was the lead of the HTA Unit in the Department of Health in the Philippines; WI is the director of the Centre for Excellence in Economic Analysis Research (CLEAR) at St Michael’s Hospital, University of Toronto and is a senior researcher at HITAP. YT conceived of the article. YT and SD wrote the first draft of the article with inputs from LCK and DB on Indonesia and the Philippines, respectively. LCK, DB, and WI reviewed and provided comments on the draft. YT and SD finalized the article. YT is the guarantor of the article.

Provenance and peer review: Commissioned; externally peer reviewed.

This article is part of a series commissioned by *The BMJ*. Open access fees are paid for by the Bill and Melinda Gates Foundation, which had no role in the decision to publish.
